# The Invisible Controller: A Decade-Long Missed Diagnosis of HIV in a Socially Vulnerable Elite Controller

**DOI:** 10.7759/cureus.93561

**Published:** 2025-09-30

**Authors:** Hafiz Fadl, Nicolas Bakinde, Minnie Mitchell, Keyera Ashe

**Affiliations:** 1 Internal Medicine, Morehouse School of Medicine, Atlanta, USA; 2 Internal Medicine, Grady Memorial Hospital, Atlanta, USA

**Keywords:** antiretroviral therapy, elite controller, functional hiv cure, hiv aids, hiv transmission risk, public health equity, severe mental illness, social determinants of health, undiagnosed hiv, viral suppression

## Abstract

Elite controllers (ECs) are rare individuals living with HIV who maintain viral suppression without antiretroviral therapy (ART). Their unique immune responses have contributed significantly to HIV cure research. However, in populations burdened by psychiatric illness and housing insecurity, such cases may go undetected, raising important ethical, clinical, and public health concerns. We present the case of a 62-year-old African American woman with schizophrenia, bipolar disorder, traumatic brain injury, and unstable housing who was hospitalized after a ground-level fall. During routine testing, she was found to be HIV-positive with a CD4 count of 1267 cells/mm³ and an undetectable viral load, despite denying any knowledge of an HIV diagnosis. A review of past records revealed an HIV-positive result from 2015, with no evidence of follow-up, disclosure, or treatment, indicating a decade-long missed opportunity. Despite never receiving ART, she met criteria for elite controller status, highlighting the intersection of rare immunologic phenomena and profound inequities in HIV detection and care. The patient’s intersecting vulnerabilities - including race, gender, homelessness, psychiatric illness, and inconsistent healthcare engagement - conspired to keep her diagnosis undisclosed for years, vulnerable to disease progression and unknowingly transmitting HIV. In an era pursuing an HIV cure, the identification of such individuals is not merely a research priority but an ethical imperative for advancing public health equity.

## Introduction

Elite controllers (ECs) are people living with HIV (PLWHIV) who maintain undetectable viral loads and normal or near-normal CD4 counts without antiretroviral therapy (ART). Although they represent less than 1% of all PLWHIV, they provide essential insights into immune regulation and viral latency [1,2]. When ECs are part of socially marginalized populations, their diagnosis may be delayed or missed entirely. Social determinants of health - including race, gender, socioeconomic status, housing, and access to mental health services - can obscure even remarkable immunologic phenomena [3,4]. These same factors can also limit opportunities for research participation, further widening health disparities [5].

In this case, the patient’s severe mental illness, including schizophrenia and bipolar disorder, further complicated her clinical course. These conditions are frequently linked to lower health literacy, higher rates of missed appointments, and stigma-related provider bias, all of which contribute to fragmented care [6]. The limited documentation of her psychiatric history, evaluation, and treatment reflects broader systemic shortcomings in mental health services. Such gaps can delay the recognition of EC status and hinder consistent engagement in care, with significant consequences for both patient outcomes and public health.

## Case presentation

This case pertains to a 62-year-old African American woman with a history of hypertension, seizure disorder, remote traumatic brain injury, bipolar disorder, schizophrenia, depression, and chronic housing insecurity who presented to the emergency department after a ground-level fall due to chronic right knee instability. She denied loss of consciousness or head trauma.

**Figure 1:**
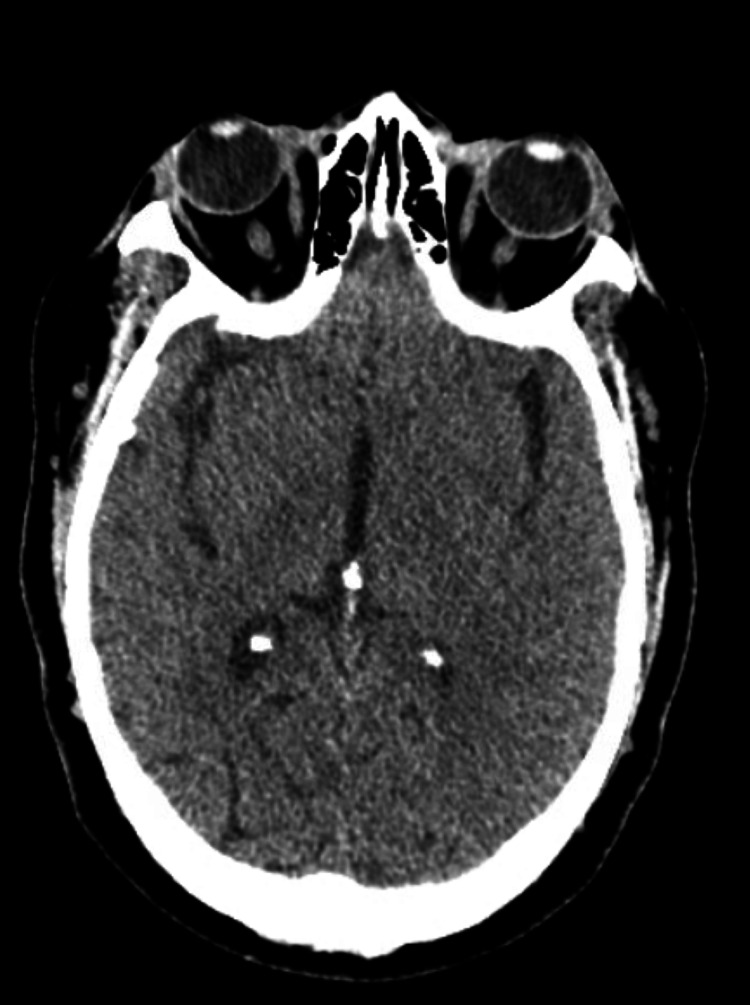
Non-contrast CT of the head. Non-contrast CT of the head showing no acute intracranial abnormalities. No acute intracranial hemorrhage or displaced cranial fracture.

She had multiple prior ED visits for falls, psychiatric decompensation, and medication refills but lacked an established primary care provider. On admission, her neurological exam was unremarkable. CT imaging of the brain showed no acute pathology (Figure 1).

Her outpatient medications included amlodipine 10 mg daily, levetiracetam (Keppra) 1500 mg twice daily, carbamazepine XR (Tegretol XR) 200 mg twice daily, vitamin D 50,000 units weekly, and vitamin B12 1000 mcg daily, although adherence was inconsistent, largely due to housing insecurity and fragmented care.

Psychiatry was consulted after she reported suicidal ideation. Evaluation revealed chronic suicidal ideation without acute intent, psychosis, or mania, likely related to depression, traumatic brain injury, and housing instability. With temporary shelter arranged, she was deemed safe for discharge with outpatient follow-up and social support referrals.

**Figure 2:**
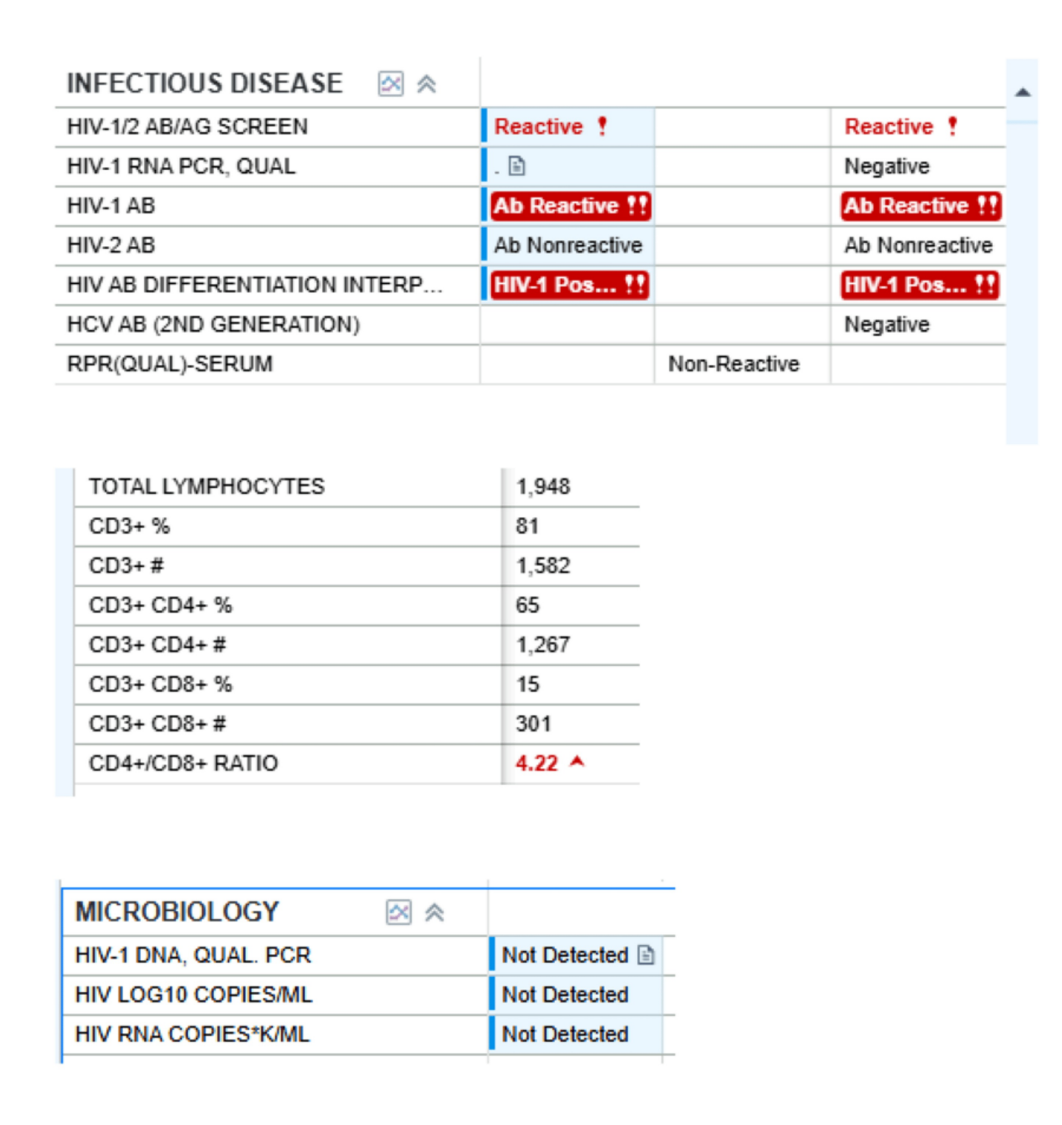
HIV-1/2 Serologic and Molecular Testing Results Laboratory findings showing HIV-1 seropositivity, CD4 preservation, and undetectable HIV RNA.

**Figure 3:**
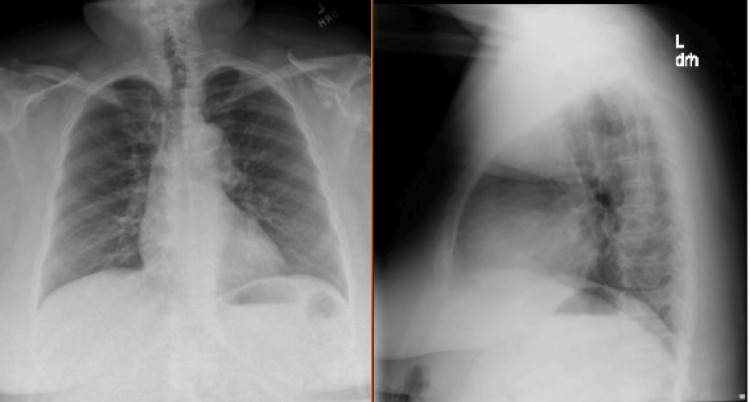
Chest X-ray (posteroanterior and lateral views) Chest X-rays include a posteroanterior (PA) view on the left and a lateral view on the right. The radiographs show clear lung fields without focal consolidation, interstitial infiltrates, cavitary lesions, or nodularity, with no evidence of opportunistic pulmonary infection.

Routine laboratory screening during admission revealed a positive HIV test, a CD4 count of 1267 cells/µL, and an undetectable HIV RNA (Figure 2). Chest X-rays show clear lung fields without focal consolidation, interstitial infiltrates, cavitary lesions, or nodularity, with no evidence of opportunistic pulmonary infection (Figure 3). She expressed surprise and denied prior knowledge of infection. A review of records from multiple health systems showed HIV positivity since 2015, without evidence of ART initiation, counseling, or documented acknowledgment of her diagnosis. Her HIV-related laboratory history is summarized in Table 1.

**Table 1:** Timeline of Key Laboratory and Diagnostic Findings. HIV RNA levels listed as “Undetectable” were below the detection threshold of the assay. Missing CD4 data are indicated with (-). “Elite control” refers to spontaneous viral suppression in the absence of antiretroviral therapy. HIV-positive status was confirmed at diagnosis and remained consistent throughout the observation period.

Test Date Notes	CD4 Count (cells/µL)	HIV RNA (log10 copies/mL)	HIV Status	Clinical Notes
January 1, 2015	-	Undetectable	Positive (confirmed)	Initial diagnostic test
January 2, 2017	1073	Undetectable	Positive (confirmed)	Viral suppression maintained
October 19, 2017	976	Undetectable	Positive (confirmed)	Continued immune stability
January 1, 2025	-	Undetectable	Positive (confirmed)	Confirmed elite control
January 5, 2025	1131	Undetectable	Positive (confirmed)	Routine follow-up
February 5, 2025	1105	Undetectable	Positive (confirmed)	Consistently preserved immunity
May 5, 2025	1267	Undetectable	Positive (confirmed)	Mild increase in CD4 count
August 22, 2025	-	Undetectable	Positive (confirmed)	Most recent viral load assessment

## Discussion

This patient’s presentation is remarkable for both her rare immunologic phenotype and the systemic inequities that delayed recognition of her status for nearly a decade. Elite controllers are of significant interest in HIV research because they can provide insight into mechanisms of natural viral suppression, including robust HIV-specific cytotoxic T lymphocyte responses, smaller viral reservoirs, and protective human leukocyte antigen (HLA) alleles [7]. These mechanisms are central to advancing functional cure strategies [8].

Her case underscores a critical health equity gap and illustrates the impact of multiple social determinants of health. She is an African American woman - a demographic that experiences disproportionately high HIV rates in the United States - where disparities in testing, linkage to care, and retention are well documented [9]. Gender-related barriers further compound these disparities, as women living with HIV often face greater delays in diagnosis and initiation of treatment [9].

Housing insecurity disrupted continuity of care and hindered coordinated follow-up. Without a stable address or reliable means of contact, healthcare providers and public health systems face significant challenges in maintaining long-term engagement and treatment adherence [10].

Her severe mental illness, including schizophrenia and bipolar disorder, added another layer of complexity. These conditions are associated with lower health literacy, higher rates of missed appointments, and stigma-related provider bias, all of which contribute to fragmented care [11]. In the absence of robust systems to re-engage and re-educate patients with psychiatric conditions, life-changing diagnoses may remain undisclosed or forgotten.

From a public health standpoint, undetected elite controller status is not without risk. While elite controllers typically have undetectable viral loads by standard assays, ultrasensitive testing can detect low-level viremia [12] and transmission remains possible [13]. Notably, transient viral rebound following immune stimulation, such as SARS-CoV-2 vaccination, has been documented in an elite controller, suggesting that virologic suppression may not be absolute under certain physiological stressors [14]. Without counseling and preventive measures, individuals in this situation could unknowingly transmit HIV, posing a public safety concern.

In addition, whether antiretroviral therapy should be initiated in elite controllers remains an important clinical consideration. Although ART is not routinely prescribed in such cases, emerging evidence suggests potential benefits, including reduction of immune activation and decreased cardiovascular risk, balanced against the challenges of adherence and the ethical complexities of initiating life-long therapy in this unique population [15]. For marginalized patients such as the one described here, these decisions also intersect with public health implications, as broader access to ART could reduce transmission risk but requires robust support systems to overcome social and structural barriers.

Finally, her case highlights a research equity challenge. Elite controllers from marginalized backgrounds are underrepresented in cure-related studies, despite calls for greater inclusion of female elite controllers - such as the “Esperanza” and “San Francisco” patients - in research [12]. Without intentional outreach to underserved populations, cure research risks overlooking important immunologic diversity, thereby limiting the generalizability of findings and perpetuating inequities in the distribution of scientific advances.

## Conclusions

This case demonstrates that exceptional immunologic control of HIV can coexist with profound systemic neglect, and that our most “elite” patients may also be our most invisible. For nearly a decade, this patient - homeless, Black, female, and living with severe mental illness - remained unaware of her diagnosis, excluded from potentially life-enhancing interventions, and capable of unknowingly transmitting the virus.

As HIV eradication strategies advance, the failure to identify elite controllers within marginalized populations constitutes both a lost scientific opportunity and a tangible public health threat. Closing this gap demands the deliberate integration of equity-driven approaches into HIV care and research.

## References

[1] Gebara NY, El Kamari V, Rizk N (2019). HIV-1 elite controllers: an immunovirological review and clinical perspectives. J Virus Erad.

[2] Kennedy BD, Blazkova J, Justement JS (2023). Comprehensive analysis of HIV reservoirs in elite controllers. J Clin Invest.

[3] Braveman P, Gottlieb L (2014). The social determinants of health: it's time to consider the causes of the causes. Public Health Rep.

[4] Bowleg L, Malekzadeh AN, Mbaba M, Boone CA (2022). Ending the HIV epidemic for all, not just some: structural racism as a fundamental but overlooked social-structural determinant of the US HIV epidemic. Curr Opin HIV AIDS.

[5] Lightfoot M, Milburn N, Loeb Stanga L (2021). Addressing health disparities in HIV: introduction to the special issue. J Acquir Immune Defic Syndr.

[6] Chukwuma OV, Ezeani EI, Fatoye EO, Benjamin J, Okobi OE, Nwume CG, Egberuare EN (2024). A systematic review of the effect of stigmatization on psychiatric illness outcomes. Cureus.

[7] Sugawara S, Reeves RK, Jost S (2022). Learning to be elite: lessons from HIV-1 controllers and animal models on trained innate immunity and virus suppression. Front Immunol.

[8] Deng Z, Yan H, Lambotte O, Moog C, Su B (2025). HIV controllers: hope for a functional cure. Front Immunol.

[9] Thomas JP, Ballew W, Kwong MH (2024). Black women's experiences along the HIV care continuum in the United States: a scoping review. Health Equity.

[10] Reddon H, Fairbairn N, Grant C, Milloy MJ (2023). Experiencing homelessness and progression through the HIV cascade of care among people who use drugs. AIDS.

[11] Nosik M, Lavrov V, Svitich O (2021). HIV infection and related mental disorders. Brain Sci.

[12] Yuan X, Lai Y (2023). Bibliometric and visualized analysis of elite controllers based on CiteSpace: landscapes, hotspots, and frontiers. Front Cell Infect Microbiol.

[13] Killian MS, Vyas GN, Mehta R, Young K, Ebrahim O (2012). Possible transmission of human immunodeficiency virus-1 infection from an elite controller to a patient who progressed to acquired immunodeficiency syndrome: a case report. J Med Case Rep.

[14] Di Girolamo L, Ferrara M, Trevisan G (2023). Transient plasma viral rebound after SARS-CoV-2 vaccination in an exceptional HIV-1 elite controller woman. Virol J.

[15] Ruiz-Mateos E, Poveda E, Lederman MM (2020). Antiretroviral treatment for HIV elite controllers?. Pathog Immun.

